# Re-Evaluation of the Critical Concentration for Ethambutol Antimicrobial Sensitivity Testing on the MGIT 960

**DOI:** 10.1371/journal.pone.0108911

**Published:** 2014-09-26

**Authors:** Sara Christianson, Dylan Voth, Joyce Wolfe, Meenu K. Sharma

**Affiliations:** 1 National Reference Centre for Mycobacteriology, National Microbiology Laboratory Public Health Agency of Canada, Winnipeg, Manitoba, Canada; 2 Department of Medical Microbiology, University of Manitoba, Winnipeg, Manitoba, Canada; Tulane University, United States of America

## Abstract

The critical concentration (CC) for ethambutol testing on the Bactec MGIT 960 *M. tuberculosis* susceptibility testing has been questioned in recent publications. In this study, we correlate susceptibility results from the Bactec 460, MGIT 960 and *emb*B gene sequencing to determine if the Bactec MGIT 960 adequately detects ethambutol resistance. We discovered discrepancies between the methods that highlight a need to re-evaluate ethambutol susceptibility testing recommendations, namely by considering lowering currently recommended CC on the MGIT 960. Further studies on the clinical significance of low-level ethambutol resistance are also required.

## Introduction

Ethambutol is one of the four primary antibiotics used in the treatment of *Mycobacterium tuberculosis* (MTB) infections and, as such, is included in the routine first line antibiotic sensitivity testing (AST) recommended by the Clinical Laboratory Standards Institute (CLSI) [1]. Though agar proportion has traditionally been considered the standard method of AST for MTB, the CLSI recommends the use of commercially available automated broth-based AST systems to facilitate faster detection of resistance. The Bactec 460 (B460) AST system (Becton Dickinson and Company (BD)) was one of the first such systems approved for testing by the United States Food and Drug Administration and agar proportion-equivalent critical concentrations (CCs) for anti-tuberculosis antibiotics were well established on that system. In accordance with the CLSI guidelines the B460 CC for ethambutol equivalent to the agar proportion CC of 5.0 ug/mL is 2.5 µg/mL. Ethambutol can also be tested at a second-line concentration of 7.5 ug/mL on the B460 (equivalent to 10.0 ug/mL using agar proportion) [1]. The B460 system has been phased out by the manufacturer and replaced by the Bactec MGIT 960 (M960).

Upon the transition from the B460 to its replacement system, the M960, CCs of the first-line antibiotics were re-assessed to ensure a good correlation between results from agar proportion, the B460 and the M960. Despite some minor reproducibility issues [2], [3], a CC of 5.0 µg/mL was established for ethambutol on the M960 to correspond with the agar proportion CC of 5.0 µg/mL and B460 CC of 2.5 µg/mL. This is the concentration of antibiotic provided in the M960 primary drug sensitivity kit provided by BD. There is no M960 equivalent to the second-line ethambutol CC provided in North America. Recent publications have challenged the M960 critical concentration of 5.0 µg/mL suggesting that it does not correlate well with alternate testing methods and is potentially unable to detect lower-level resistance that is still clinically relevant [4]–[7].

In addition to phenotypic AST, molecular testing for resistance has become routine for rapid detection of antibiotic resistance. Mutations at 3 locations in the *emb*B gene (amino acid positions 306, 406 and 497) have a very high correlation with phenotypic resistance [8]–[12]. Mutations at these three locations are generally considered to be good predictors of ethambutol resistance, though they are not found in all ethambutol resistant strains of MTB.

## Methods

Thirty two isolates previously determined to be resistant to ethambutol at 2.5 µg/mL using the B460 and ten ethambutol-sensitive MTBC isolates were selected from the NRCM culture collection. The B460 and M960 (where available) ethambutol sensitivity data was recorded for these strains and the isolates were anonymized prior to testing for this study. Random study numbers were applied in the following manner; isolates resistant to the CC of 7.5 µg/mL on the B460 were assigned a number beginning with “H”, those resistant at 2.5 µg/mL but sensitive at 7.5 µg/mL on the B460 were assigned a number beginning with “L” and those that were sensitive on the B460 were assigned a number beginning with “S” (Table 1). Isolates were tested to determine ethambutol resistance/sensitivity at the recommended CC of 5.0 µg/mL using the M960 and screened for *emb*B 306, 406 and 497 mutations as previously described [12]. The results from this analysis did not correlate as expected with the B460 results. A number of strains that were resistant to ethambutol on the B460 were either not identified as resistant on the M960, or gave inconsistent results upon the confirmation of resistance by repeat testing. Due to the presence of *emb*B mutations associated with resistance in many of the strains, MIC data for ethambutol from the M960 was obtained to clarify the discrepancies. Isolates were tested at ethambutol concentrations of 1.0 µg/mL, 2.0 µg/mL, 4.0 µg/mL, 5.0 µg/mL, 8.0 µg/mL, 10.0 µg/mL, and 16.0 µg/mL according to the manufacturer’s instructions. Ethambutol powder was provided either from the BD SIRE kit or Sigma-Aldrich (St. Louis, MO).

**Table 1:** *emb*B mutations and Bactec MGIT MIC values for study isolates.

SpecimenNumber	*emb*Bmutation	Bactec MGIT MIC(µg/mL)
H1	Met306Val	16.0
H2	Met306Val	>16.0
H3	no mutation	8.0
H4	Met306Val	8.0
H5	Met306Val	10.0
H6	Gly406Asp	5.0
H7	Met306Val	8.0
H8	Met306Val	16.0
H9	Met306Val	16.0
H10	Gly406Ala	4.0
H11	Met306Val	>16.0
H12	Met306Val	10.0
H13	no mutation	10.0
H14	Met306Val	16.0
H15	no mutation	<5.0
H16	Gln497Arg	4.0
L1	no mutation	4.0
L2	no mutation	5.0
L3	Gly406Ser	5.0
L4	no mutation	4.0
L5	no mutation	4.0
L6	no mutation	4.0
L7	no mutation	4.0
L8	Gly406Ala	4.0
L9	Met306Val	16.0
L10	Met306Ile	4.0
L11	Met306Leu	10.0
L12	Gln497Arg	4.0
L13	Met306Ile	4.0
L14	Met306Ile	4.0
L15	Met306Ile	4.0
L16	Met306Ile	4.0
S1	no mutation	<1.0
S2	no mutation	2.0
S3	no mutation	<1.0
S4	no mutation	<1.0
S5	no mutation	<1.0
S6	no mutation	<1.0
S7	no mutation	<1.0
S8	no mutation	2.0
S9	no mutation	<1.0
S10	no mutation	2.0

## Results and Discussion

All phenotypically sensitive strains based on B460 testing were also sensitive on the M960 with MIC values of < = 2.0 µg/mL to ethambutol. None of these strains had mutations at any of the three locations most commonly associated with resistance in the literature (Table 1).

Sixteen of the 32 ethambutol resistant strains were resistant to both the high and low CC of ethambutol on the B460. Twelve of these had an MIC of > = 8.0 ug/mL on the M960, while two had an MIC of 4.0 µg/mL and two had an MIC of 5.0 µg/mL. Three of the latter 4 isolates had mutations at positions 406 (Gly406Asp, Gly406Ala) and 497 (Gln497Arg) of *emb*B indicating a high possibility of resistance, yet they were sensitive at the M960 CC of 5.0 µg/mL (Table 1).

The remaining sixteen resistant isolates were resistant at the lower CC of 2.5 µg/mL ethambutol on the B460, but sensitive at the higher B460 CC. Two of these isolates had MICs on the M960 of 10 and 16 µg/mL. These two isolates had mutations of Met306Leu and Met306Val respectively. The 14 remaining isolates had MIC values between 4.0 and 5.0 µg/mL. Five of these 14 strains had Met306Ile mutations, one Gly406Ser mutations, one Gly406Ala mutation and one Gln497Arg mutation. Six strains had no EmbB 306, 406 or 497 mutations (Table 1).

There were a total of 18/32 ethambutol resistant isolates with MICs values of 4 or 5 µg/mL on the M960. Three of these strains performed inconsistently at the M960 CC of 5 µg/mL, initially showing resistance at 5.0 µg/mL, but the results were not reproducible. Thirteen of these 18 (72%) had EmbB 306, 406 or 497 mutations. All 18 of these isolates were identified as resistant using the previous CC of 2.5 µg/mL on the B460. Based on this information, a CC of <5.0 µg/mL on the M960 would more accurately identify strains with, what appears to be, a lower level of resistance to ethambutol, and more accurately emulate the results from the B460.

Ultimately, 15/32 (46.8%) isolates that were identified as resistant by the B460 were not identified by the M960 CC of 5.0 µg/mL leading to a sensitivity of only 62.5%. The *emb*B mutation data supports the “resistant” designation by the B460 in 62.5–81.3% of cases (depending on level of resistance). Additionally, 72% of resistant cases with an MIC for ethambutol in the 4.0–5.0 µg/mL range had mutations that help to confirm resistance. Mutations particularly associated with this level of resistance were Met306Ile, Gly406 mutations and Gln497 mutations.

## Conclusions

Multiple explanations have been put forth for discrepancies between ethambutol sensitivity testing methods [3], [7]. Our study points to the inability of current M960 testing methodology to detect resistance in isolates with ethambutol MIC values very close, or equal, to the CC. These results highlight a need to re-evaluate both the methodology for determining ethambutol resistance on the M960 as well as the need to determine the clinical significance of lower-level ethambutol resistance. Unfortunately, at the present time there is limited data available on the clinical significance of low-level ethambutol resistance. As proposed by Gumbo (2010) and Sirgel *et al* (2012) [4], [5], a slight lowering of the M960 CC for ethambutol may help ensure that resistance is not under-reported. Further investigation of these issues through studies correlating low-level ethambutol resistance with clinical outcomes of ethambutol treatment is required to determine if a change in sensitivity testing methodology is warranted.
